# An ex-vivo assessment of a new single probe triple modality (Trilogy) lithotripter

**DOI:** 10.1007/s00345-022-04127-8

**Published:** 2022-08-24

**Authors:** Charles Joseph O’Connor, Donnacha Hogan, Lee Chien Yap, Louise Lyons, Derek Barry Hennessey

**Affiliations:** Department of Urology, Mercy University Hospital, Cork, Ireland

**Keywords:** Trilogy, Settings, Begostone, Percutaneous, Nephrolithotomy

## Abstract

**Introduction and objectives:**

This Swiss LithoClast^®^ Trilogy lithotrite is a new lithotrite for percutaneous nephrolithotomy (PCNL). It has four modifiable settings; impact, frequency, ultrasound and suction. We aim to determine the optimal device settings for the fastest stone clearance.

**Materials and methods:**

Kidney stone phantoms were made with Begostone in a powder to water ratio (15:3–15:6). Complete stone clearance (seconds) was calculated and impact and frequency were adjusted and repeated *N* = 3. Intra renal pressure (IRP) was then measured in a porcine kidney model.

**Results:**

Stone phantoms with physical properties similar to struvite were cleared best with 100% impact and frequency of 12 Hz. Both uric acid stone phantoms and calcium phosphate stone phantoms were cleared most efficiently with an impact of 30% and a frequency of 4 Hz. The mean time to clear uric acid stone phantoms was 83 s versus 217 s for calcium phosphate stone phantoms. Similarly, for calcium oxalate stone phantoms, an impact of 30% and a frequency of 4 Hz was associated with the fastest clearance time, mean 204 s. However, the differences between 4, 8 and 12 Hz were not statistically significant. At a suction level of 60% or higher, IRP became negative.

**Conclusion:**

These results indicate that stone phantoms of hard kidney stones are cleared more efficiently at lower impact and frequency settings. With regard to suction, a setting of ≤ 50% appears to be the optimal setting.

## Introduction

Percutaneous nephrolithotomy (PCNL) is the preferred treatment for renal stones larger than 20 mm in diameter [1, 2]. Many devices have been used for stone removal during PCNL and were termed lithotrites. Early lithotrites in PCNL used compressed air to accelerate a projectile inside a hand piece, which hit a probe to elicit a shock wave that moved through the probe to the stone to cause fragmentation (ballistic lithotripsy). Other single probe lithotrites used piezoceramic crystals to creating ultrasonic waves that caused the probe’s tip to vibrate and achieve fragmentation (ultrasonic lithotripsy) [3]. These fragments were then removed by a grasper.

Dual-energy single probe lithotrites were developed, combining ballistic lithotripsy and ultrasound lithotripsy. While this was a modernisation, a downside was that these devices were mounted within one another. This resulted in reduced stone clearance speeds. Another modification was the addition of suction to the probe, increasing stone clearance, but devices could only use this with a single fragmentation modality, i.e. ballistic and suction or ultrasonic and suction only [4–6]. The latest generation of lithotrite is called the LithoClast^®^ Trilogy (Electro Medical Systems S.A., Nyon, Switzerland). This device has combined ultrasonic lithotripsy, ballistic lithotripsy and suction capability in single probe. Several different probe sizes are also available. Studies have suggested that the Trilogy offers faster stone clearance than other ultrasonic and combination ultrasonic devices [3, 7–9].

The Trilogy lithotrite has four modifiable settings; impact (0–100%), frequency (2–12 Hz), ultrasonic lithotripsy (0–100%) and suction (0–100%). The exact combination of these variables for the most efficient stone clearance of different stone types is unknown. The objective of this study was to determine the optimal settings (impact and frequency) for four of the most common kidney stone compositions. The secondary aim was to determine the effect of different suction settings on intrarenal pressure (IRP) in porcine kidney model to determine the optimal suction setting.

## Methods

### Artificial kidney stone production

Begostone (BEGO USA, Lincoln, USA) was mixed in ratios of 15:3, 15:4, 15:5 and 15:6 of powder to sterile water. In these ratios, 15:3 was comparable to calcium oxalate monohydrate (COM), 15:4 to calcium phosphate (CaP), 15:5 to uric acid and 15:6 to struvite [10]. Begostone and water were mixed for at least 60 s until the resulting mixture was homogeneous. The mixtures were then placed on a contact vibrator to remove air bubbles. The resulting mixture was poured into 2 cm^3^ silicone moulds and allowed to sit for at least 8 h. An ultrasonic flaw detector (KRAUTKRÄMER USM GO + , Waygate Technologies, Ahrensburg, Germany) was used to measure the longitudinal wave speeds for each stone type. Results were then compared to the known physical properties of kidney stones.

### Determination of intrarenal pressure in conventional PCNL with Trilogy

Whole intact urinary tracts were harvested from Landrace pigs slaughtered for the food chain by a licenced veterinarian. Three well dissected porcine kidneys were used for testing to ensure comparable results and validity. A 5Fr cystometry abdominal pressure line connected to an external strain gauge was placed into the renal pelvis and sutured in place with a purse-string suture. The intrarenal pressure was then calibrated to zero representing atmospheric pressure. Pressure readings were recorded using calibrated cystometry software. The pig kidney was punctured and dilated to 26Fr, and a 26Fr Amplatz sheath was placed. A 26Fr nephroscope was then placed into the kidney. A 3L bag of saline was set at 100 cm and 60 cm above the level of the kidney with the irrigation fluid channel fully open on the scope. The Trilogy lithotripter was then placed into the kidney via the nephroscope and the effect of the suction from the device on IRP was determined.

### Determination of optimal settings for stone clearance

**Fig. 1:**
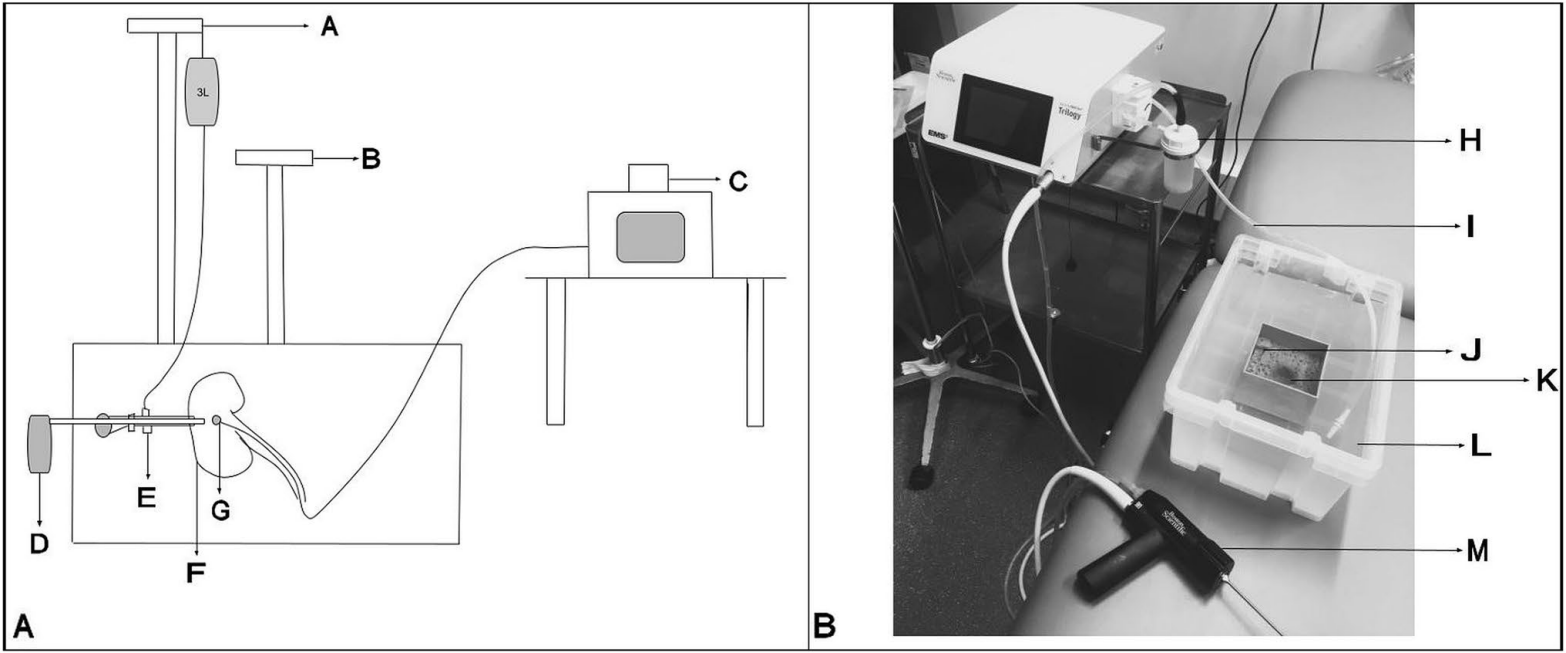
Set up of experiments. **A** IRP measurement: A = 100 cm IV stand with irrigating fluid, B = 60 cm IV stand, C = cystometer, D = Trilogy handpiece, E = nephroscope, F = porcine kidney, G = pressure transducer. **B** Stone clearance measurement: H = filter, I = filter outflow tubing, J = inner sponge, K = small plastic container, L = water bath, M = Trilogy handpiece

A water bath was filled with normal saline 0.9%. A fixed inner container was filled with sponge. This sponge had a cylindrical shape carved from the centre. This held a small soft plastic container that contained 2 cm^3^ artificial kidney stones. This set up is shown in Fig. 1B. All of these stones were soaked in water for 2 mins prior to testing. The outflow from the filtered suction fluid was secured in the water bath, so the saline level in the system remained constant. Trilogy probes of 3.4 mm × 340 mm were used. As per manufacturers’ guidelines, suction and ultrasound were kept constant at 40% and 100%, respectively. Impact and frequency were adjusted for a combination of 9 different settings. The 9 different settings used were 30% impact and 4 Hz frequency, 30% and 8 Hz, 30% and 12 Hz, 60% and 4 Hz, 60% and 8HZ, 60% and 12 Hz, 100% and 4 Hz, 100% and 8 Hz and 100% and 12 Hz. Each setting was repeated *N* = 3 for the four different artificial kidney stone mixes. The primary outcome was time to total stone clearance. This outcome was measured by a single operator using a consistent technique. The suction tubing, filter, probe and handpiece were cleared of stone fragments between runs to ensure testing conditions remained constant. Time was also taken to make sure the suction tubing was clear of blockages during runs.

### Statistical analysis

Statistics were calculated using SPSS. All data were normally distributed and therefore given as mean (± standard deviation). An Independent *t* test was used to compare mean values for normally distributed data. One-way ANOVA (Turkeys multiple comparison) was used when three or more independent variables were compared. A *p* value of < 0.05 was considered statistically significant.

## Results

### Comparison of artificial kidney stone parameters to known parameters of kidney stones

Begostone and water when mixed in a ratio of 15:3 and set into a stone phantom had comparable physical properties to that of COM kidney stones. The longitudinal wave speeds by ultrasonic flaw detector were similar 4201 ± 27 m/s for the stone phantom vs 4535 ± 58 m/s for COM stones measured by Zhong et al. [10]. Density was also similar 2056 ± 6 kg/m^3^ vs 2038 ± 34 kg/m^3^. All percentage differences between stone phantoms and actual kidney stones in terms of longitudinal wave speed and density were less than 10%, except for the density of CaP stones and longitudinal wave speed of struvite stones. CaP stone phantoms were 15.4% more dense than actual kidney stones, while struvite phantoms had a longitudinal wave speed 15.8% higher than actual struvite stones.

### Intrarenal pressure in conventional PCNL with a suction lithotripter

The Trilogy Lithotripter with a 3.9 mm × 340 mm probe was inserted into a pig kidney via a 26Fr sheath and 26 nephroscope. The change in IRP was assessed at different suction settings (0–100%) at both 60 cm and 100 cm irrigation heights. Suction strengths ≥ 60% resulted in a negative IRP. IRP was significantly lower using a 60 cm irrigation height compared to 100 cm at all suction strengths. At an irrigation height of 100 cm, IRP was 22 cmH_2_0 at 20% suction, 15.25 at 30%, 8.5 at 40% and 3.25 at 50%. At a height of 60 cm, IRP was 11.8 cmH_2_0 at 20% suction, 8.6 at 30%, 3.6 at 40% and 0.5 at 50%.

### Optimal frequency and impact for the clearance of artificial kidney stones

**Fig. 2:**
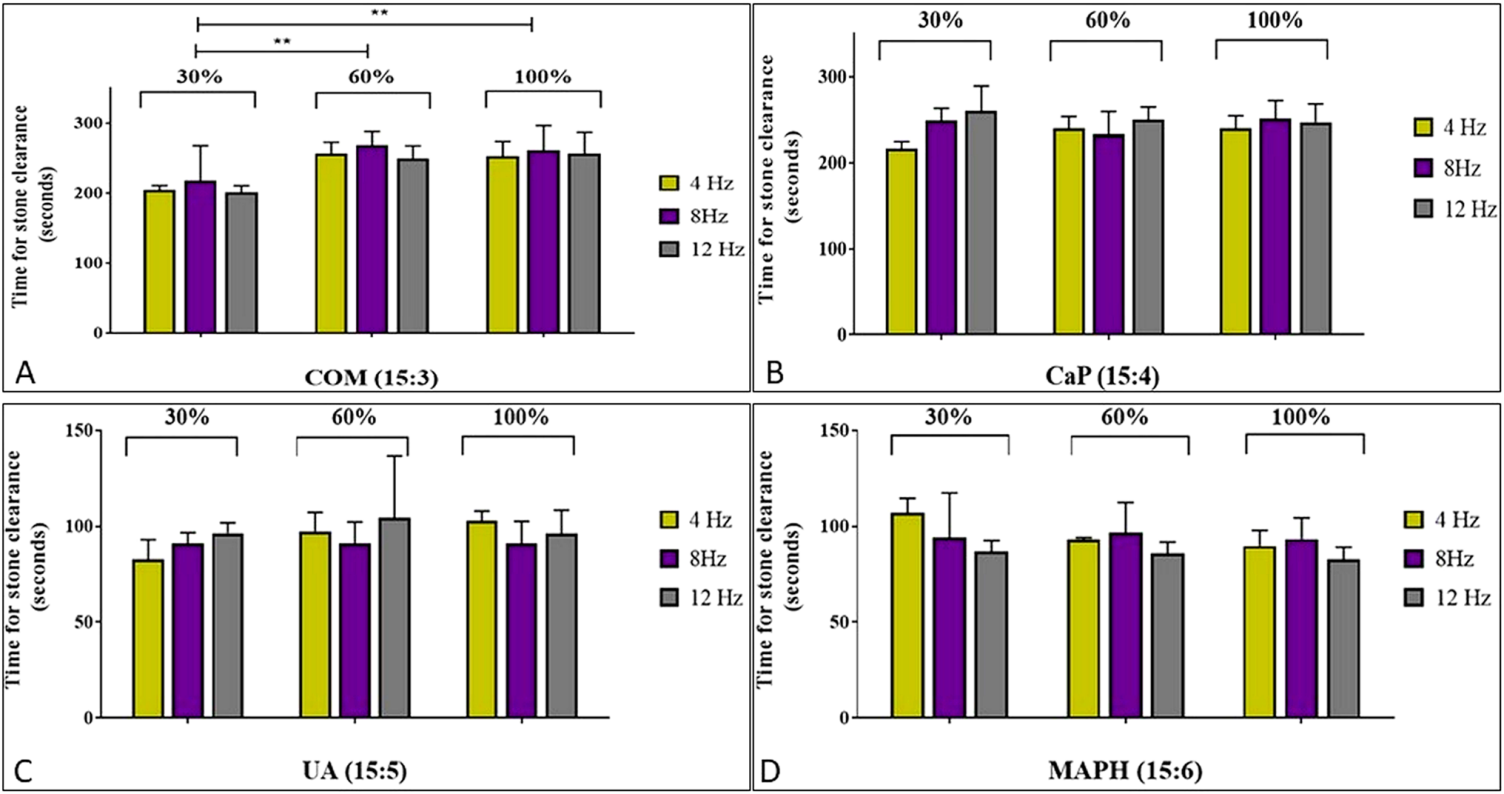
Effect of varying frequency and impact of total stone clearance time for the different stone phantoms. *COM* calcium oxalate monohydrate, *CaP* Calcium phosphate, *Hz* hertz (frequency). Statistical significance between lithotripsy settings assessed using ANOVA (Tukey's multiple comparison). **p < 0.001

The fastest total stone clearance time for COM stone phantoms was with an impact of 30% and frequency of 4 Hz (mean 204 s). A clearance rate of 571.42 mm^3^/min was noted. The slowest stone clearance time for COM phantoms was 60% and 8 Hz (mean 269 s or 446.10 mm^3^/min). Reducing the impact from 100 to 60% and 30% resulted in significantly faster stone clearance rimes (*p* < 0.001). Reducing the Hz appeared to have a lesser effect. Data are shown in Fig. 2A. CaP phantoms were cleared fastest at the 30% impact and a frequency of 4 Hz (mean 217 s), while the slowest stone clearance was at the 30% and 12 Hz setting (mean 261 s). These findings were similar to COM stone phantoms where reducing the Hz appeared to have a lesser effect. Data are shown in Fig. 2B. Uric acid phantoms were also fragmented fastest at the 30% impact and 4 Hz frequency (mean 83 s or 1445.79mm^3^/min). This was similar to COM and CaP phantoms but fragmented times were faster. 60% and 12 Hz was the slowest setting for breaking up this stone type (mean 104 s). Data are shown in Fig. 2C. In contrast to the other stone phantoms, Struvite phantoms fragmented most efficiently at the 100% impact and 12 Hz. Mean clearance time of 83 s. The slowest setting for struvite stone clearance was at 30% and 4 Hz (mean 108 s or 1111.11 mm^3^/min). Interestingly, this setting was the fastest for the other stone types. Data are shown in Fig. 2D. The average time to total stone clearance across the nine settings and four stone types was (889.8125 mm^3^/min ± 227.40 SD:454.93).

## Discussion

Experientially and clinically the Trilogy has performed excellently at kidney stone fragmentation and removal [11]. However, to date there has been no study to determine the optimal settings for different kidney stones types. This is the first study to our knowledge that aims to determine the best settings for the most efficient stone clearance for four of the most common stone types.

For hard kidney stones phantoms (calcium oxalate monohydrate and calcium phosphate), the fastest total stone clearance times were associated with low impact and low frequency. Uric acid is considered an intermediate stone with regard to hardness. Similar to COM and CaP, the fastest stone clearance times were seen with low impact and low frequency. In contrast to the other kidney stones, struvite, a soft stone, was cleared most efficiently with the highest impact and frequency settings.

These results are somewhat unexpected and are the inverse to the predicted results. The lowest impact and frequency settings tested for hard and intermediate stones were the fastest stone clearance times. For soft stones, the highest impact setting and highest frequency resulted in a fast clearance time. We theorised that higher impact and frequency settings were potentially associated with the reduced transfer of ultrasonic energy due to a reduced probe to stone contact time associated with these. Lower energy settings also result in reduced dispersion of stone fragments, allowing the Trilogy’s suction to function more effectively. Struvite stones broke in to larger fragments during testing due to the stone’s lower tensile failure strength [12]. This minimised the dispersion effect of high energy settings. This may explain the efficiency of high impact and frequency settings in this group.

Overall fragmentation times of Begostone in this ex-vivo study were significantly longer than those described in other benchtop studies, such as those by Carlos et al. and Bader et al. These two studies placed 1 cm^3^ Begostone stone phantoms of the 15:3 consistency (calcium oxalate monohydrate) in hemispherical silicone supports in a water bath for testing. Carlos et al. calculated an average total time to stone clearance of (23.79 s or 2522.07 mm^3^/min) while Bader et al. calculated an average of (26 s or 2307.87 mm^3^/min) [3, 8]. Across our nine different settings for calcium oxalate, the average time to total stone clearance was (240.77 s or 498.4 mm^3^/min ± 17.67). The likely explanation for this was using a different, soft plastic container, allowing for greater dispersion of stone fragments. We felt this better represented a stone sitting in a renal pelvis. We do not believe this is a difference in the efficacy of the Trilogy between studies but a difference in experimentation setup.

The average stone clearance rate across all of the experiments in this study was (889.81 mm^3^/min ± 227.46). This stone clearance rate is quite similar to the in-vivo mean stone clearance rate recorded in a European multicentre prospective study on behalf of the European section of UroTechnology. This study included data from 157 PCNL procedures and calculated the mean stone clearance rate as 65.55 mm^2^/min or 945 mm^3^/min based on calculated 3D volume [11]. Nottingham et al. recorded a more efficient stone clearance rate to Thakare et al. of 68.9 mm^2^/min, while another prospective study by Sabnis et al. recorded an average stone clearance rate of 590.7 ± 250 mm^3^/min [13, 14]. A multi-institutional prospective randomised controlled trial by Large et al. calculated stone clearance rates across 51 PCNL procedures using the Trilogy. Their mean clearance rate was (1220 mm^3^/min ± 1670). [9]

The suction effect on IRP during PCNL with The Swiss Lithoclast^®^ Trilogy suction is also an unknown variable. Suction can vary from 0 to 100% on the consoles touch screen. Our data show that negative pressure is generated at suction strengths above 60%, and the renal pelvis collapses. This causes loss of vision intra-operatively and can also lead to bleeding within the renal pelvis. The author's opinion is that suction strengths above 60% are unlikely to be required for PCNLs while using the larger probe sizes (3.4 mm/3.9 mm). A suction strength of 30–40% is likely optimal to maintain an IRP so that vision is not impaired and fragments are removed. The effect of suction on IRP is intuitive and supported by a 2017 study by Abourbih et al., which found that increasing the suction on a nephroscope was more effective than making two tracts in a porcine model [15]. Another interesting finding from the data shown is that increasing suction allows an increase in the height level of irrigating fluids while maintaining a safe IRP; this thus allows the operator to speed up irrigation safely. A similar study on IRP in a porcine model found the optimal suction for PCNL with the Trilogy to be 10% to maintain a constant pressure within the kidney [16].

This study does have some limitations. These experiments were conducted in controlled conditions using a single-operator and consistent technique. However, human and device errors cannot be disregarded. Human error in this experiment consisted of subtle changes in the fragmentation of artificial kidney stones, with some stones fragmenting in a more physically favourable manner. Steps taken to reduce this error included ensuring the Trilogy’s device tip was in constant contact with the stones and ensuring the device and tubing were clear of fragments during and between runs. Another limitation was that a single probe size was used. However, we feel this size is representative of all large Swiss Lithoclast^®^ Trilogy probes.

These findings have changed practice in our institution and intra-operative stone clearance times have improved subjectively. The authors are currently in the process of recruiting patients for a trial to assess the efficiency of these settings in clinical practice.

## Conclusion

This experiment shows that with harder stones, lower frequency and impact settings may result in more efficient fragmentation. The 30% impact function was proven to fragment calcium oxalate monohydrate stones most efficiently. After investigating the effect of the Trilogy's suction on IRP, the investigators conclude that suction of ≤ 50% is optimal.

## Figures and Tables

**Fig. 3:**
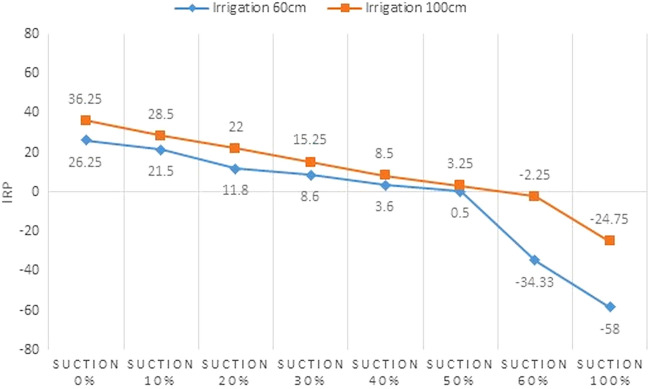
Trilogy suction settings and the effect on intrarenal pressure. *IRP* intrarenal pressure (cmH_2_0), C*m* centimetres

## Appendix

See Table 1 and Fig. 3.

**Table 1:** Comparison of physical characteristics of artificial kidney stones to real kidney stones

Begostone powder to water ratio	CL (m/s)	% Difference CL (m/s)	P (Kg/m^3^)	% Difference P (Kg/m^3^)	Kidney stone type	CL (m/s) [10]	P (Kg/m^3^) [10]
15:3	4201 ± 27	7.4	2056 ± 6	0.9	COM	4535 ± 58	2038 ± 34
15:4	3815 ± 0	3	1825 ± 34	15.4	Cap	3932 ± 134	2157 ± 16
15:5	3374 ± 48	2.8	1717 ± 61	10	Uric acid	3471 ± 62	1546 ± 12
15:6	3325 ± 90	15.8	1753 ± 29	9.5	Struvite	2798 ± 82	1587 ± 68

CL (m/s); longitudinal wave speed. P (Kg/m^3^); density.
